# Effects of Valence and Emotional Intensity on the Comprehension and Memorization of Texts

**DOI:** 10.3389/fpsyg.2019.00179

**Published:** 2019-02-01

**Authors:** Olga Megalakaki, Ugo Ballenghein, Thierry Baccino

**Affiliations:** ^1^CRP-CPO (EA7273), Université de Picardie, Amiens, France; ^2^CHART/LUTIN (EA 4004), Université Paris 8, Paris, France

**Keywords:** emotions, arousal, comprehension, memorization, adults

## Abstract

In the present study, we independently manipulated valence (positive, negative, or neutral) and emotional intensity (low, medium, or high), asking what impact they have on text comprehension (via surface, paraphrase, and inference questions) and memorization (via Remember/Know test) in adults. Results show that emotional contents, including valence and intensity affects comprehension. Emotional valence had a significant effect on text comprehension, with higher scores for positive and neutral texts than for negative ones. Participants scored higher on the surface questions for positive texts and on the inference questions for negative texts, with equivalent scores for paraphrase questions. Regarding emotional intensity, medium intensity generally fostered better comprehension of both positive and negative texts. High emotional intensity is beneficial for positively valenced texts, but hinders the understanding of negatively valenced ones. Regarding memorization, participants recalled more emotional words than neutral ones, and more words for positive texts than for either negative or neutral ones. In conclusion, our results show that emotions play an important role and improve the processing of information.

## Introduction

The past 20 years have seen growing research interest in the link between emotions and cognition (for a review see Kaakinen et al., 2018). Studies have shown that emotions are involved in judgment and decision-making, and should therefore be regarded as an integral part of cognitive functions (Damasio, 1994; Frijda, 2010). We set out to improve current understanding of how the emotion conveyed by a text, and more specifically its valence (i.e., degree of pleasantness) and intensity (i.e., degree of arousal), affects the comprehension and memorization of that text.

Up to now, studies of the link between emotions and text comprehension have either examined the effect of inducing of an emotional state in the reader beforehand, using emotionally charged material (Ziegler, 2014; Niemiec and Lachowicz-Tabaczek, 2015), or explored readers’ construction of a representation of the main protagonist’s emotional status (i.e., Gernsbacher et al., 1992; Gygax et al., 2003; Gillioz et al., 2012; Mouw et al., 2017). In the present study, we focused on emotions conveyed by the whole text rather than focusing on the main protagonist.

Today, it is widely accepted that we retain information better if we judge it to be important. This raises the question of whether emotional valence contributes to judgments of importance. According to LeNy (1979), each text possesses a *semantic relief* that is determined both by the importance of the information and its emotional charge. These two components can also be dissociated, as it is possible to have important information that is weakly affective, and information of little importance that is strongly affective. In their model of text comprehension, Van Dijk and Kintsch (1983) postulated that emotions can guide readers in their creation of a coherent representation of the situation described by the text. Emotional feelings drive readers’ attention and help them to decide which information needs to be activated and which information is relevant to the situation. According to Van Dijk and Kintsch (1983), emotions therefore constitute an active control structure. This selection role was also postulated by Miall (1989), who claimed that emotions indicate to readers what is important and guide the content of their mental representations. Kneepkens and Zwaan (1994) discussed the artifact emotions that arise from the structure of the text and lead readers to engage in more effective processing. This makes the text’s structure easier to memorize, with readers remembering not only the emotions induced by the text, but also the object of these emotions.

In one of her studies, Davidson (2006) showed that, for children aged 6-10 years, the emotions experienced by the protagonists of a story favor the memorization of the events associated with these emotions. According to Davidson (2006), the emotion induced by an event makes the latter more salient, but the emotion must be felt by the child if the event is to be represented in memory. Blanc (2010) was interested in the comprehension of stories featuring different types of events (spatial, causal, temporal, emotional) in children aged 5-7 years. She expected the emotional dimension to be one of the best tracked dimensions, along with the causal dimension, and predicted that explicit emotional information (words designating the emotion) in the tale would be better remembered than that requiring inferences to be drawn (characters’ reactions or emotion-inducing events). Results revealed, however, that the children did not treat the emotional dimension as a prerequisite for comprehension. Instead, they tended to rely on two other dimensions (causal and spatial). Nevertheless, as soon as they had fully assimilated the causal dimension and acquired a good understanding of emotions, the latter became key to comprehension (Blanc, 2010).

Readers’ ability to represent protagonists’ emotions was explored by Gernsbacher et al. (1992). Participants read short stories that each described the goals and actions of one main character, and their consequences for the life of a second character. The emotional state of the main character was explicitly disclosed after the story, in a target sentence, and the authors manipulated its coherence (match or mismatch) with the context of the story. The authors postulated that if the readers understood the main character’s emotional state, they would read the target sentence more quickly if it contained a matching emotion word. If the readers had not formed any representation of the emotional state, there would be no difference between the sentences containing matching vs. mismatching emotion words. Results showed that when the target sentence mentioned an emotional state that did not match the situation described in the story, it was read more slowly. Moreover, in a second experiment, Gernsbacher et al. (1992) showed that this occurred independently of the valence (positive vs. negative) of the emotional state, as they observed an increase in reading times even when the mismatched emotion word had the same valence as the emotional state described in the story. When Gygax et al. (2004) asked participants to read stories followed by sentences containing either a specific emotion, a synonym of that emotion, or an emotion that was broadly similar, they found no differences in reading times for the different sentences, indicating that the representations of the character’s emotion constructed by readers were not specific to one emotion word (e.g., *sad*), but were more generic, encompassing several different but matching terms. Results by Gillioz et al. (2012) support the idea that what readers deduce during reading are more the behaviors associated with emotions than the emotions themselves (*per se*). Afzali (2013) also showed that emotions can improve cognitive processes. In her study, 79 Persian-speaking students, divided into two groups, had to read four stories, each in a different genre (horror, romance, humor, or detective), and answer 10 questions to check the emotions triggered by the texts. Finally, participants had to complete a post-test of 20 items for each text, to measure their comprehension of the stories. The experimental group, who learned to engage their emotions during reading by writing and talking about their feelings, performed better on the comprehension post-tests. Thus, the results of this study indicate that involving emotions while reading has a beneficial influence on students’ understanding of short literary texts.

### Process Positive or Negative Information

Researchers generally agree that *emotional valence* also plays a role in comprehension, so how does its impact on this cognitive activity vary according to whether it is *positive or negative?* Guéraud and Tapiero (2001) looked at the effect of the valence of retrieval cues on comprehension. Six narrative texts were constructed with the same organization: an introduction containing the retrieval cue (positive, negative or neutral connotation); a goal game with two versions (goal satisfied vs. not satisfied); and an intermediate episode where the final sentence contained the same retrieval cue as before. Two target sentences were then presented, to analyze reactivation of the goal. Participants read the texts and answered comprehension questions. The authors predicted that the valence of the retrieval cue would influence the activation spread and reactivation of information, with negatively connoted information constituting a more effective retrieval cue than positive or neutral information. In the nonsatisfied goal condition, a negatively connoted cue would result in longer reading times than a positively connoted one, while the latter would in turn give rise to longer reading times than a neutral cue. Key findings indicated that, in this condition, participants actually had longer reading times when the cue was neutral, rather than positive or negative. The presence of emotional information seemed to increase the processing speed, compared with neutral information, and serve as a cue for information retrieval. Readers seem quite sensitive to this type of information. In a study conducted among pupils in their last year of primary school, Clavel and Cuisinier (2010) showed that the most insightful children when it came to identifying the emotional experience of the protagonist in a story (taken from children’s literature) were also the ones who achieved the best comprehension scores, but only when the story had a negative emotional valence. In the same direction go the results of Davidson (2006) who observed that the valence of emotions influences memorization. Events are better remembered when they are related to negative, rather than positive, emotions. By contrast, Ducreux-Fournier (2007, Unpublished) found that negative emotional induction had a detrimental effect on text comprehension. These divergent results can be explained by the types of texts that were used, as well as by their emotional intensity.

### Role of Emotional Intensity to Process Information

Emotional valence is therefore not the only factor that needs to be taken into account. *Emotional intensity* also influences text comprehension, even if there has been little research on this aspect. According to Frijda et al. (1992), this lack of interest stems from the sheer difficulty of measuring the intensity of the emotion, as this depends on the individual, the context, the goals, and the value of the event. These authors posit that the emotional process has several components (e.g., cognitive processes, physiological variations) that each contribute to emotional intensity. These components are related to different dimensions, and can be evaluated in many different ways. Frijda et al. (1992) therefore prefer to talk about *emotional dimensions* rather than emotional intensity. They showed that perceived intensity varies according to five independent dimensions: the memory of the event, the duration of the emotion, the importance of bodily variations, behaviors, and action tendencies. The same idea of dynamic and cumulative emotional components is defended by Scherer (2005), who defines emotion as a process of coordinating information in five organismic subsystems (information processing, support, executive, action, and monitoring), starting with the appraisal of an event, be it internal or external. Each subsystem is associated with a particular component of emotion (i.e., cognitive, psychophysiological, action tendency, motor expression, or subjective feeling), and it is the combination of all these component changes that results in a specific emotion.

Early work on emotional intensity focused on the memorization of words and found that a message with an affective tone (pleasant or unpleasant) is remembered better than a neutral message (Dutta and Kanungo, 1975). Other more recent studies have manipulated the intensity of emotional terms (Schmidt, 2012). Concerning text comprehension, in a recent study, Mouw et al. (2017) examined how children and adults process negative emotions while reading target sentences, and how they rate their own emotional states and those of the protagonists. The authors used an offline measure to assess participants’ ratings of the valence and arousal dimensions of emotional states, in addition to sentence reading times. Results indicated that reading times were shorter, and processing was easier, when the sentences described negative emotions. In a series of studies, Martins (1982, 1993) showed that the emotional intensity of information must be taken into account in addition to its level of importance. In one experiment, Martins (1982) asked a group of participants to read a newspaper article about the Nazis during the Second World War in order to answer questions on it. Results showed that a negative valence and high emotional intensity contribute to the process of memorizing and retrieving information. In another experiment, Martins (1993) measured the recall of six descriptive texts whose emotional content had been manipulated (three very moving and three not very moving), having previously shown that the importance of the information was not correlated with its emotional intensity. Results showed that participants recalled the information in the text more according to its importance than according to its emotional intensity. The influence of the latter was only observed when the text was very moving. This confirmed the findings of Legros (1988), who used two versions of the same newspaper article that differed solely in their emotional intensity (weakly vs. strongly emotional, featuring terms with stronger affective connotations). He presented these versions to two groups of high-school students aged 16-18 years. Participants in the strongly emotional condition achieved better recall performances, suggesting that texts need to be very emotionally intense to foster comprehension. However, in another study, Denhière and Legros (1983) obtained different results. In their experiment, two texts about the murder of a youth by a security guard were presented with different emotional connotations (weaker vs. stronger). The authors expected the highly emotional text to be more easily recalled, but the main findings indicated that the weakly affective text tended to be better remembered than the strongly connoted one.

Observations in the literature of differences in performance according to the text’s emotional valence can be seen in relation to the models of unitary memory put forward by Anderson (1983, 1993) and Cowan (1998), as well as the theory of depth of encoding (Tulving, 2002). In their activation theories, Anderson (1983, 1993) and Cowan (1998) tackle the relationships between working memory and long-term memory by considering working memory to be the active part (attentional focus) of long-term memory. For these authors, working memory is not a separate structure, but a long-term internal memory process that allows some of the information to be activated. According to their theories, cues activated during reading can be used to retrieve the meaning of a story. In addition, according to the encoding specificity principle (Tulving, 2002), recall is facilitated if specific cues present during encoding are also present at retrieval. Emotional cues, like syntactic or semantic cues, therefore play an important role in both encoding and retrieval, and determine the depth of memorization.

### Scope and Hypotheses

As we have just seen in the literature review, the involvement of emotions in cognitive processes, and more especially in text comprehension, is a widespread and largely accepted idea today. Thus the valence and emotional intensity of a text influence its comprehension and improve performances. However, in most previous studies, these two dimensions (valence and intensity) were closely interwoven, making it impossible to pinpoint the role of each dimension. Thus, in the present study, we independently manipulated valence and emotional intensity, asking what impact they have on text comprehension and how they interact in adults. We took short ecological texts, and varied their valence (positive, negative, or neutral) and emotional intensity (low, medium, or high). To probe the participants’ text comprehension, we asked them questions of differing depth: surface, paraphrase and inference (Kintsch et al., 1990). We also looked at the impact of emotional words on memory, using the Remember/Know (R/K) paradigm (Tulving, 1985).

Our *first hypothesis* was that emotional valence influences text comprehension. We expected to observe higher scores when the questions were posed for emotional, rather than neutral, texts. This is why emotional information is treated differently from neutral information (Kitayama and Howard, 1994) because emotions inform the individual of potential hedonic consequences (Pratto, 1994). Concerning the type of question, we expected that participants respond better for surface questions than for paraphrase ones, and better performances for paraphrase questions than for inference ones.

We then focused on the effects of valence (positive or negative) and intensity (low, medium, or high) in text comprehension. We expected to observe better scores for the negatively valenced texts than for the positively valenced ones, as research in the literature shows that texts associated with negative emotions give rise to an easier processing of the events (Davidson, 2006; Mouw et al., 2017). Also we expected better scores for the highly emotional texts than for the less emotional ones like previous works where participants in the strongly emotional condition achieved better recall performances (Legros, 1988). We also expected to observe an interaction effect between valence and intensity for emotional texts and a greater interaction effect for negative and high-intensity texts (*Hypothesis 2*).

Our *third and final hypothesis* concerned the R/K recognition test. We predicted that, regardless of the text’s valence and emotional intensity, participants would recall more emotional words than neutral words, and would be more confident in their recollection n the case of emotional words. Jacobs (2015) suggested that it is possible to evaluate the potential that a text has for inducing an emotional response in the reader based on the valence of the individual words used in it. The higher the text’s intensity, the better participants would remember the emotional words they had read. Finally, they would remember words better if they came from a negatively valenced text rather than a positively valenced one, and the effect of intensity would be more marked for negatively valenced texts than for ones with a positive valence as research shows that texts associated with negative emotions give rise to a better memorization (Davidson, 2006).

## Materials and Methods

### Participants

Participants were 31 psychology students from the University of Picardie (26 women), with a mean age of 21.90 (*SD* = 3.94). All participants had French as their mother tongue, no reading impairment, and normal or corrected-to-normal (with contact lenses) vision. This study was carried out in accordance with the recommendations of French law written informed consent from all subjects. All participants gave written informed consent in accordance with the Declaration of Helsinki. Full review and approval was not required according to our Institution’s guidelines and national regulations.

### Text Construction

The texts used in our study were written by adult volunteers native French speakers, mean age was 22.28 years (SD 3.21) on a website specially constructed for our study. All participants had a high-school degree and an average of 2.58 years of college education (SD 0.56). They were instructed to write a short (800-1000 characters), easily understandable and emotional text that triggered strong feelings (positive or negative). They then had to say what feelings were described in their stories, and rate the intensity and emotional valence of each other’s texts on a 9-point nonverbal Likert-like scale featuring figurines, known as the Self-Assessment Manikin (SAM; Lang, 1980). For the purposes of our experiment, emotionally neutral filler stories were also constructed, in order to compare the emotional texts with neutral ones. Through this protocol, we were able to collect 120 texts, divided into different categories according to their valence and intensity. If participants circled a figurine between 1 and 4 on the valence scale, we considered the text to be negative. By contrast, if they surrounded a figurine between 6 and 9, we categorized the text as positive. If they surrounded the fifth figurine, we considered the text to be neutral. With regard to the texts’ emotional intensity, we divided the scale into three parts: 1-3 (low), 4-6 (medium), and 7-9 (high).

We then asked 12 different (but similar to the experimental sample) adult volunteers native French speakers, mean age was 22.9 (SD 1.9), 7 male and 5 female, to perform *triple ratings* (valence, emotional intensity, and comprehensibility) of the texts, again using the SAM. Participants had a high-school degree and an average of 3.1 years of college education (SD 1.4).

After calculating interrater agreement (valence: Cronbach’s alpha = 0.95; arousal: α = 0.97; comprehensibility: α = 0.95), we selected 75 texts that were all easily understandable, but varied in valence and emotional intensity.

These 75 texts were divided into different categories in terms of valence and intensity (negative valence with low, medium or high intensity, and positive valence with low, medium or high intensity). We then selected randomly 18 of these texts with comprehensibility greater than 6 over 7: six negative and six positive texts with three intensities (two low, two medium, and two high), and six neutral texts. The Table 1 below presents the descriptive statistics for texts and in the Appendix two examples of the texts: one high arousal negative text and one high arousal positive text.

**Table 1:** Descriptive statistics for texts: Mean (SD).

Valence arousal	Positive			Negative			Neutral
	Low	Medium	High	Low	Medium	High	
Mean number of characters	1002.5(4.95)	1009.5(31.82)	966(48.08)	977(30.41)	969.5(38.89)	1023(27.57)	1067(110.16)
Mean number of words	166.50(0.71)	173(1.41)	170.50(7.79)	171(14.14)	171.5(17.68)	187.50(14.85)	172(10.95)
Mean Comprehensibility	6.54(0.53)	6.58(0.47)	6.50(0.35)	6.58(0.23)	6.87(0.18)	6.33(0.12)	6.69(0.41)
Arousal (SAM scale)	[1–3]	[3–6]	[6–9]	[1–3]	[3–6]	[6–9]	5
Valence (SAM scale)	[6–9]			[1–3]			5


*Valence and Arousal: [1–3]: 1 and 3 are included. [3–6]: 3 and 6 are excluded.*

*Comprehensibility: 12 judgements on a Likert scale from 1 (not comprehensible) to 7 (very comprehensible).*

### Comprehension Test

To assess their text comprehension, participants had to respond to six questions (two surface, two paraphrases, and two inferences) probing three levels of representation (Van Dijk and Kintsch, 1983; Kintsch et al., 1990; McNamara et al., 1996; Penttinen et al., 2013; Megalakaki et al., 2016; Porion et al., 2016). The surface level is a literal representation and corresponded to the memorization of the words or sentences as they appeared in the text, but not their meaning. The paraphrase (textbase) level corresponded to the memorization of the meaning of each sentence, as well as of the whole text. As for the inference (situation model) level, this involved linking the information provided by the text to the information already in memory, via inferences. More precisely, text comprehension inferences are conceived of as semantic integrations. Furthermore, both textbase and situation model levels are considered semantic representations derived from the text.

### Memory Test

To gauge the impact of the emotional words on memory, we administered a test based on the R/K paradigm (Tulving, 1985) that assesses the nature, depth and quality of recall. We used 20 words for each text: five neutral words and five emotional words taken from the text, plus 10 fillers (five neutral and five emotional). To achieve a more objective choice of emotional words, we relied on Bonin et al. (2003) and the Emotaix database (Piolat and Bannour, 2009). For the neutral texts, we used 10 words taken from the text and 10 fillers. For each of the 20 words, participants had to answer “yes” or “no” to the question “Did you read this word? If YES, do you remember it in detail?” More specifically, participants who answered “yes” had to indicate whether they had a clear recollection of the word in the text (R) or just a feeling of familiarity (K). We assumed that R responses would reflect the use of episodic memory to retrieve encoding details (Besson et al., 2012). Analyses were based on the percentage of correct R responses and correct K responses.

### Procedure

After participants had been informed about the terms of the study and signed the consent form, they were provided with an iPad 2 Air 9.7 (resolution: 2048 ^∗^ 1536 pixels) and written instructions. Their comprehension of the instructions was verbally verified. Participants could read each single-page text (double-spaced, in Times New Roman, 12-point font size) at their own pace, moving through it by swiping the screen to the left. They were not allowed to return to previous screens. The experiment began with a training phase featuring a single text. Participants then read the 18 experimental texts (order of presentation randomized across four groups). After reading each text, participants had to orally answer the six questions and provide written responses for the R/K test. The oral responses were collected by the experimenter and recorded directly in an excel document during the experiment. The whole experimental session lasted approximately 1 h.

### Scoring and Data Analysis

For the comprehension test, each text was associated with 2 surface questions, 2 paraphrase questions, and 2 inferential ones. Our raw scoring was to score 1 for a correct answer and 0 for a wrong answer. Thus, we obtained average scores for each type of text (e.g., score for positive text with low arousal for surface questions = number of correct answers/4).

For the R/K test, we calculated the proportions of correctly recognized items with recollection (pRcib) [(*n*^scorepRcib^/10 ^∗^ 100) = %], and correctly recognized items with no recollection (pKcib) [(*n*^scorepkcib^/10 ^∗^ 100) = %] (Besson et al., 2012, p. 249).

Statistical analyzes were carried out using Statistica 10 (Statsoft.fr). We carried out repeated measures ANOVAs in order to inspect main effects of valence and arousal. Then, we conducted planned comparisons analyses in order to determine the significant differences between the different valences and levels of arousal.

For the comprehension test, the analysis that has been done comparing the level of understanding according to the type of question (surface, paraphrase, inferential) and texts’ valence (neutral, positive, negative). In this analysis, neutral is considered as a valence, like positive or negative.

In order to analyze the influence of the arousal intensity, we then analyzed emotional texts only. We conducted a 2 (valence: positive, negative) × 3 (intensity: low, medium, high) × 3 (type of question: surface, paraphrase, inference) ANOVA to compare the positive and negative texts. Both valence and intensity were within-participants factors. Regarding performances on the R/K test, we ran a 2 (valence: positive, negative) × 3 (intensity: low, medium, high) × 2 (Recall: R, K) × 2 (emotional nature of words recalled: neutral, emotional) ANOVA.

## Results

### Comprehension

There was a main effect of type of question (surface, paraphrase, inference), *F*(2, 60) = 61.28, *p* < 0.001, η^2^_*p*_ = 0.67. The mean percentage of correct answers was significantly higher for surface questions (84%, *SD* = 15.18) than for paraphrase ones (70%, *SD* = 15.17), and significantly higher for paraphrase questions than for inference ones (63%, *SD* = 14.86). Planed comparisons analyses revealed significant differences between surface and paraphrase questions (*p* < 0.001), surface and inference questions (*p* < 0.001), and paraphrase and inference questions (*p* < 0.05).

The *interaction between type of question and text valence* was also significant, *F*(4, 120) = 11.82, *p* < 0.001, η^2^*_*p*_* = 0.28. Planned comparisons showed that participants answered surface questions less correctly for negative texts than for positive or neutral ones, *F*(1, 30) = 45.04, *p* < 0.001. No significant difference was found between the three emotional valences for paraphrase questions. Finally, participants answered inference questions better when they were about negative texts than when they were about positive or neutral ones, *F*(1, 30) = 9.34, *p* < 0.001. Moreover, for inference questions, there was a significant difference between negative and positive texts, *F*(1, 30) = 12.87, *p* < 0.001, but not between positive and neutral or negative and neutral texts (*p* > 0.05). In general, type of question had no impact on the rate of correct answers for negative texts, and the same pattern was observed for positive and neutral texts, namely, lower response rates for surface, paraphrase and inference questions.

In order to analyze the influence of the arousal intensity, we analyzed emotional texts only. So next, we focused on the *emotional texts (positive and negative)* and the effects of *valence* and *emotional intensity* on text comprehension. Analysis failed to reveal any major effect of emotional intensity on mean scores, *F*(2, 60) = 0.92, *p* = *ns*. However, an interaction effect was found between valence and emotional intensity, *F*(4, 120) = 2.71, *p* < 0.05, η^2^*p* = 0.08. Planned comparisons showed that for high-intensity texts, there was a significant difference between mean comprehension scores for negative and positive texts, *F*(1, 30) = 13.21, *p* < 0.01. These mean scores were significantly higher for positive texts (74%, *SD* = 25.93) than for negative ones (68%, *SD* = 22.56).

An interaction effect was also revealed between type of question and emotional intensity, *F*(4, 120) = 3.048, *p* < 0.05, η^2^*p* = 0.09. While there were no differences between the three intensity levels for the surface and paraphrase mean scores, there was a significant difference between medium and high intensity for the inference questions, *F*(1, 30) = 4.76, *p* < 0.05. For low intensity, there was a significant difference between surface and paraphrase mean scores, *F*(1, 30) = 20.866, *p* < 0.001, as well as between both paraphrase and inference mean scores, *F*(1, 30) = 14.012, *p* < 0.001, and surface and inference mean scores, *F*(1, 30) = 82.869, *p* < 0.001. For medium intensities, mean scores were significantly higher for paraphrase questions than for either surface questions, *F*(1, 30) = 16.831, *p* < 0.001, or inference questions, *F*(1, 30) = 16.167, *p* < 0.001. By contrast, for high-intensity texts, mean scores were significantly higher for surface questions than for paraphrase questions, *F*(1, 30) = 39.262, *p* < 0.001, but there was no significant difference between paraphrase and inference questions, *F*(1, 30) = 0.061, *p* = *ns*. Mean scores on inference questions were higher for high-intensity texts than for texts of either low or medium intensity.

**FIGURE 1:**
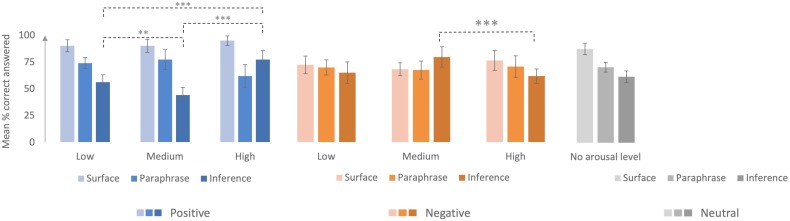
Mean percentage of correct answers, depending on valence, intensity and type of question. Error bars denote 95% confidence intervals. ^∗∗^*p* < 0.01; ^∗∗∗^*p* < 0.001.

Analysis of the triple interaction between valence, intensity and type of question revealed differences between the intensities of positively valenced texts for responses to paraphrase questions (low-medium vs. high, *F*(1, 30 ) = 9.72, *p* < 0.01), as well as for responses to inference questions (low vs. medium, *F*(1, 30) = 9.62, *p* < 0.01, low vs. high, *F*(1, 30) = 15.51, *p* < 0.001, and medium vs. high, *F*(1, 30) = 60.96, *p* < 0.001). No significant differences were found for surface questions (Figure 1).

For negative texts, we only found one difference for the inference questions (medium vs. high intensity, *F*(1, 30) = 15.08, *p* < 0.001), with poorer performances for high-intensity texts.

### Remember/Know Task

**FIGURE 2:**
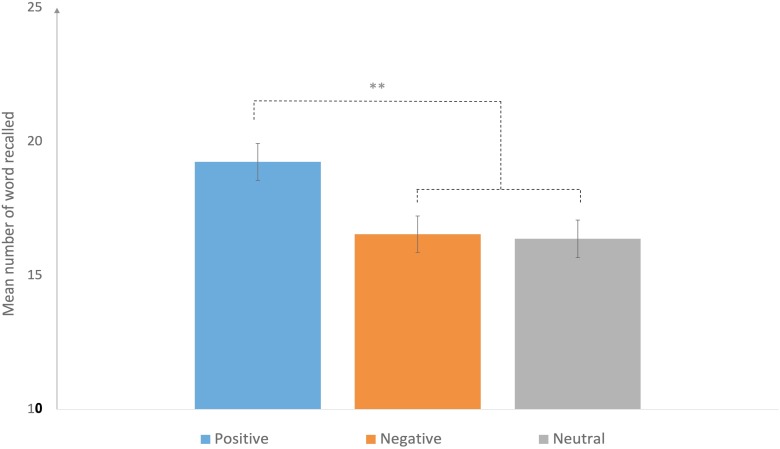
Mean number of correctly recalled words, depending on the emotional valence of the texts. Error bars denote 95% confidence intervals. ^∗∗^*p* < 0.01.

Analysis reveal a *main effect of the nature of the recall* on the number of correct responses, with significantly more R responses than K responses, *F*(1, 30) = 36.61, *p* < 0.01, η^2^*p* = 0.92. There was also a main effect of the emotional nature of the word (emotional vs. neutral). Participants recalled significantly more emotional words than neutral words, *F*(1, 30) = 69.69, *p* < 0.01, η^2^*p* = 0.96. We also observed a main effect of the texts’ emotional valence (positive vs. negative vs. neutral), as the mean number of recalled words was significantly higher for positive texts than for either negative or neutral ones (Figure 2). Planned comparisons confirmed this significant result, *F*(1, 30) = 25.68, *p* < 0.01. No significant difference was observed between negative and neutral texts (*p* > 0.05).

We demonstrated an interaction effect between the emotional nature of the recalled words and R/K performances, *F*(1, 30) = 13.53, *p* < 0.05, η^2^*p* = 0.68. There were more R responses for emotional words than for neutral ones, *F*(1, 30) = 41.80, *p* < 0.01, whereas there were no significant differences in K responses, *F*(1, 30) = 1.56, *p* = *ns*.

There was also an interaction effect between the emotional nature of the recalled words and the *valence* of the texts, *F*(1, 30) = 12.19, *p* < 0.01, η^2^*p* = 0.91. There were no significant differences in the recall of emotional words between positive and negative texts, *F*(1, 30) = 5.62, *p* = *ns*, whereas neutral words were significantly better recalled when they came from positive texts rather than from negative ones, *F*(1, 30) = 107.58, *p* < 0.01.

There was no significant difference in the effect of emotional intensity between positive and negative texts.

**FIGURE 3:**
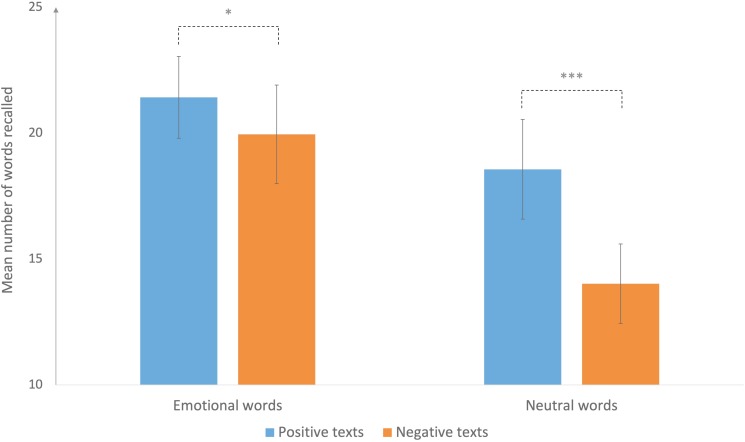
Mean number of correctly recalled words, depending on word type and the emotional valence of the texts. Error bars denote 95% confidence intervals. ^∗^*p* < 0.05; ^∗∗∗^*p* < 0.001.

The interaction between the emotional valence of the texts (positive vs. negative) and the emotional (or otherwise) nature of the recalled words was also significant, *F*(1, 30) = 40.35, *p* < 0.01, η^2^*p* = 0.91. More emotional words were recalled when they came from positive texts rather than from negative ones, *F*(1, 30) = 7.29, *p* < 0.05. We also found a significant difference for neutral words, *F*(1, 30) = 7.29, *p* < 0.001, which were better recalled when they came from positive texts rather than from negative ones (Figure 3). There was no interaction between valence and emotional intensity.

## Discussion

The objective of this research was to study the effects of valence and emotional intensity on text comprehension and memorization.

Our *first hypothesis* was that emotional valence influences text comprehension, with higher scores for emotional texts than for neutral ones. We expected participants to perform better on surface questions than on paraphrase questions, with lower performances still for inference questions. Results partially validated our hypothesis, by showing that emotional valence had a significant effect on text comprehension, with higher scores for positive and neutral texts than for negative ones. Regarding the type of question (surface, paraphrase, or inference), results showed that it influenced text comprehension, with higher scores for surface questions than for paraphrase ones, and lower scores for inference questions. These results validated our hypothesis, and underlined the relevance of using these three types of questions to assess text comprehension (Van Dijk and Kintsch, 1983).

Results showed better performances for positive vs. negative texts, and contradicted previous findings of better performances for negative texts (Kitayama and Howard, 1994; Pratto, 1994; Guéraud and Tapiero, 2001; Davidson, 2006; Clavel and Cuisinier, 2010). They did not validate our *second hypothesis* about the superiority of negatively valenced texts. However, the three types of questions we asked helped us to understand the differences we observed better. Results showed that scores on surface questions were higher for positive and neutral texts, whereas the opposite was true for inference questions, with better scores for negative texts. Scores on paraphrase questions were equivalent across the three types of texts, indicating, among other things, the relevance of the texts we used in terms of their comprehensibility. Our results suggest that the emotional valence of a text plays an important role in its understanding. A positively valenced text promotes the comprehension of its content and structure, whereas a negatively valenced one favors inferences. The higher performances on *surface questions* for *positive texts* can be attributed to a more detailed memorization of the text’s content and the sequence of events that participants discovered as they moved through the text. We can think that participants pay more attention to the unfolding of all the events described in the story and thus memorize more surface information. For negative texts, we can postulate that it was the overall value of the negative event and the context in which it took place that was best retained, explaining the superiority of performances on the inference questions, which related the events described in the text to the readers’ prior knowledge. We can view these results in the light of Kitayama and Howard (1994)’s model of amplification, which occurs in activation processes and attentional orientation, and can be explained by the importance of emotional information for our survival. In the case of negative texts, the emotion results in a restriction of the individual’s attentional field, resulting in a focus on the main aspects, to the detriment of more peripheral ones (Easterbrook, 1959; Loftus, 1979). These results also corroborate Spachtholz et al. (2014)’s observation that, relative to neutral affect, negative affect reduces individuals’ working memory capacity, but increases their accuracy, and may improve the quality of memories.

Regarding the impact of emotional intensity on text comprehension, we expected to observe better performances for high-intensity texts than for ones of lower intensity. Results did not show any main effect of emotional intensity, but there was a difference between negative and positive high-intensity texts. There were no differences for low- or medium-intensity texts. This result suggests that high emotional intensity promotes the understanding of positive texts, but hinders that of negative texts. High intensity appears to lead to a more partial understanding of negative texts. We can assume that an event described in a negative high-intensity text constitutes an obstacle to understanding because the reader focuses on the negative elements of the text, to the detriment of the comprehension and memorization of the other information. It is also interesting to note that the high intensity of the positive texts facilitated performances on the inference questions, whereas medium intensity favored performances on paraphrase questions for the positive texts, and inference questions for the negative texts. We can once again attribute this differential impact of emotional intensity on the text comprehension to the readers’ construction of a partial representation of the emotions described in the text according to multiple other factors, including their motivations, knowledge and previous experience of the event being described (Gygax et al., 2007), and empathic abilities (Altmann et al., 2012).

Taken together, the text comprehension results show that it is medium emotional intensity that generally gives rise to better performances, both for positive texts (paraphrase questions) and for negative ones (inference questions). This indicates the importance of its use for promoting the process of text comprehension. High emotional intensity promotes the comprehension of positive texts, but hinders it for negative texts (at the level of inference questions), It would appear to do so by focusing attention on negative information, thus loading working memory and making it harder to understand the text as a whole (Denhière and Legros, 1983; Martins, 1993; Gygax et al., 2007).

Concerning the R/K task, participants gave more R responses than K responses, indicating that they clearly recollected the words in the text (Besson et al., 2012). In general, more emotional words were recalled than neutral words, suggesting a deeper memorization of emotional words and partially validating our third hypothesis. Results also showed an effect of the texts’ emotional valence, with more words (both emotional and fillers) being recalled for positively valenced texts than for either negatively or neutrally valenced ones. They therefore did not validate our hypothesis on this point, confirming instead the results of the studies showing that negative affect reduces working memory capacity (i.e., Spies et al., 1996). These results suggest that negatively valenced texts reduce attention by focusing it exclusively on negative terms, loading working memory and thus decreasing recollection. This effect has commonly been attributed to the allocation of resources to task-irrelevant thoughts, resulting in poorer performances (Spachtholz et al., 2014). The R/K results therefore point in the same direction as the results of the comprehension test, indicating that memory traces are less precise in the case of negative texts, and suggesting that a negative valence hinders the processes of memorization and comprehension. Like other authors, we attribute these results to the fact that a negative emotional valence captures more attention, decreases motivation, and leaves fewer resources for the completion of the ongoing task (Anderson, 1983, 1993; Ellis and Ashbrook, 1988; Cowan, 1998; Pekrun, 2006; Spachtholz et al., 2014). Concerning *emotional intensity*, texts of low or high intensity elicited better performances. This divergence of results from those for text comprehension underscores the difficulty of considering the contribution of emotional intensity to the comprehension and memorization of texts, as these are dynamic processes where several factors interact, including the individual, the context, the goals, and the value of the event (Frijda et al., 1992).

In sum, our results show that valence and emotional intensity play an important role in text comprehension and memorization. They corroborate previous results showing that since emotional stimuli are emotivational, they are more likely to be attended to than neutral stimuli, and undergo better processing than neutral stimuli (Lang et al., 1997). We can interpret this influence as indicating that the valence and emotional intensity of texts could constitute specific cues that are present during encoding and may contribute to deeper processing of the information (Tulving, 2002), thus facilitating subsequent recall. In general, memories acquired in one emotional state are best retrieved when one is again in the same emotional state. These specific emotional cues may correspond to the active part of working memory during the encoding phase, in line with activation theories (Anderson, 1983, 1993; Cowan, 1998). Anderson and Cowan describe working memory as the active part, at a given moment and according to the needs of the task, of long-term memory. In their models, these authors make no real distinction between working memory and long-term memory, and simply distinguish between elements that are more or less activated in memory. Those elements that are in the *attentional focus* (here, emotional cues) are more strongly activated. Thus, when individuals engage in a task involving the participation of working memory, they can activate representations stored in long-term memory.

Within this theoretical framework, we can ascribe the better performances for positive texts to the gradual activation of positive emotional cues, allowing time for the information to be taken on board as the story unfolded, without overloading working memory. Concerning the poorer performances for negative texts, be that attention may have remained focused on the negative emotional cues, overloading working memory, preventing the integration of elements of the narrative, and making comprehension of the whole text more difficult. In this case, presumably, individuals quickly drew inferences based solely on the negative emotional cues.

## Conclusion

In conclusion, our results show that emotions act as retrieval cues for the events to which they are linked. They play an important role and improve the processing of information. In this information processing, a positive emotional valence seems to facilitate understanding. However, when the type of question (surface, paraphrase, or inference) is taken into account, we find that while positive texts favor comprehension of the text’s content and structure (surface questions), negative texts favor the drawing of inferences about the overall value of the event described in the story (inference questions). The results of our study also showed that high emotional intensity is beneficial for positively valenced texts, but hinders the understanding and memorization of negatively valenced ones. Moderate emotional intensity generally seems to promote comprehension. However, it should be noted that it is difficult to accurately assess the role of emotional intensity because of its dynamic nature and the involvement of several components (i.e., cognitive, psychophysiological, action tendency, motor expression, and subjective feeling), as Scherer (2005) pointed out, with changes in these components resulting in specific emotions. To understand the influence of emotions on comprehension better, it would be interesting to assessing changes in all the components involved (Scherer, 2005). Like other authors (Scherer, 2005; Gygax et al., 2007), we believe that the embodied cognition framework provides a particularly relevant basis for future studies. Indeed, in our ongoing research, we are considering cognitive but also physiological processes (eye movements and body movements via sensors) to better understand their interactions in the processing of emotional information.

## Author Contributions

OM designed the study and wrote the manuscript. UP ran the statistical analyses and drafted the manuscript. TB designed the study and drafted the manuscript.

## Conflict of Interest Statement

The authors declare that the research was conducted in the absence of any commercial or financial relationships that could be construed as a potential conflict of interest.

## Appendix

Two examples of the texts: one High arousal Negative text and one High arousal Positive text.

**High Arousal Negative Example:**

Il était face à moi, je ne voyais que ses yeux noirs et son revolver pointé sur mon front. Un coup d’électricité parcourt mon corps, de mes orteils au plus haut de mon crâne. Des gouttes de sueur longent mon dos, ma bouche s’assèche, mon cœur s’accélère et je sens la peur me tétaniser. Mais comment aurais-je pu imaginer ce matin en me levant que ma journée tournerait ainsi ? J’aurais pu commencer par aller chez le boucher ou le poissonnier mais non, je me retrouve ici, dans cette épicerie du centre-ville avec ce type qui me serre de plus en plus fort. Il est 11h46 et ma vie est sur le point de s’arrêter. Il hurle, me traine jusqu’au comptoir, mes jambes ne me portent plus. Mon souffle est de plus en plus rapide et saccadé, des larmes roulent sur mes joues, la frayeur se lit sur mon visage. Tout est flou autour de moi et je n’arrive presque plus à distinguer ses paroles. «La caisse ou je la descends!» sont les seuls mots que j’arrive à percevoir. Lorsque le coup de feu retentit, le monde autour de moi se fige et je m’écroule.	He was facing me, I only saw his black eyes and his gun on my forehead. An electricity blast went through my body, from my toes to the top of my skull. Drops of sweat are going down my back, my mouth is dry, my heartbeats raise and I feel the fear paralyzing me. How could I imagined this morning when I woke up that my day will take that turn? I could have started to go to the butcher, or the fishmonger but no, here I am, in that grocery of the center of the town, with that guy tighting me harder and harder. It is 11h46 and my life is about to end. He screams, struggle me to the countertop, my legs barely support me. My breath is faster and faster and jerky, tears are rolling down my face, fright can be read on my face. Everything is blurred around me, and It is hard for me to ear his words. “The cash or I shot her dead!” are the only words I perceive. When the gunfire sounds, the world around me freeze, and I collapse.

**High Arousal Positive Example:**

Le bonheur de devenir grande soeur pour la première fois de sa vie, c’est un sentiment tellement magique… A cette époque je n’avais que 2 ans, je ne comprenais pas encore ce sentiment de bonheur que j’allais ressentir en te prenant dans mes bras sur le lit de maman à la maternité, ce fameux 19 novembre 1998. Tu étais toute petite et toute mignonne dans ton petit lit... Je me rappelle même avoir dit à papa qu’une fois rentrée, je voulais te mettre dans ma poussette de poupée car tu étais trop petite pour marcher. Je t’aimais déjà tellement puis par la suite, à l’âge de mes 6 ans, notre petit frère a vu le jour, pour ma part j’étais une seconde fois envahie par le bonheur et toi pour la première fois tu as pu ressentir ce besoin de protéger ce petit être... Le bonheur d’être une grande soeur. Pour rien au monde j’aimerais que vous disparaissiez de ma vie. Je vous aime tellement Oceane et Léo. Ma fierté.	The happiness of becoming a big sister for the first time in her life is such a magical feeling... At that time I was only 2 years old, I did not understand the feeling of happiness that I was going to feel when I took you in my arms on the mother’s bed at the maternity, this Wednesday, November 19, 1998. You were very small and cute in your little bed. I even remember telling Dad that when I got home, I wanted to put you in my doll stroller because you were too small to walk. I loved you so much and then, at the age of 6, our little brother was born, for my part I was a second time invaded by happiness and you for the first time you could feel this need to protect this little being... The happiness of being a big sister. For nothing in the world I would like you to disappear from my life. I love you so much Oceane and Leo. My pride.
